# The Sociological Perspective of Users’ Invisible Work: A Qualitative Research Framework for Studying Digital Health Innovations Integration

**DOI:** 10.2196/25159

**Published:** 2021-11-04

**Authors:** Dilara Vanessa Trupia, Alexandre Mathieu-Fritz, Tu Anh Duong

**Affiliations:** 1 LATTS, Univ Gustave Eiffel, CNRS, Ecole des Ponts Marne-la-Vallée France; 2 INSERM, Chaire Avenir Santé Numérique IMRB U955, Équipe 8 University of Paris-Est Créteil Créteil France; 3 AP-HP Department of Dermatology Hôpital Henri-Mondor Créteil France

**Keywords:** digital health innovations, qualitative analysis, sociological framework, invisible work, patient work, user work, participatory health care, chronic illness, self-quantification

## Abstract

**Background:**

When new technology is integrated into a care pathway, it faces resistance due to the changes it introduces into the existing context. To understand the success or failure of digital health innovations, it is necessary to pay attention to the adjustments that users must perform to make them work, by reshaping the context and sometimes by altering the ways in which they perform activities. This adaptation work, most of which remains invisible, constitutes an important factor in the success of innovations and the ways in which they transform care practices.

**Objective:**

This work aims to present a sociological framework for studying new health technology uses through a qualitative analysis of the different types of tasks and activities that users, both health professionals and patients, must perform to integrate these technologies and make them work in their daily routine.

**Methods:**

This paper uses a three-part method to structure a theoretical model to study *users’ invisible work*. The first part of the method includes a thematic literature review, previously published by one of the coauthors, of major sociological studies conducted on digital health innovations integration into existing care organizations and practices. The second part extends this review to introduce definitions and applications of the *users’ invisible work* concept. The third part consists of producing a theoretical framework to study the concept according to the different contexts and practices of the users.

**Results:**

The paper proposes four dimensions (organizational, interactional, practical, and experiential), each composed of a set of criteria that allow a comparative analysis of different users’ work according to different health technologies.

**Conclusions:**

This framework can be applied both as an analytical tool in a research protocol and as an agenda to identify less visible adoption criteria for digital health technologies.

## Introduction

### Background

A large number of digital health technologies are developed every day, each being increasingly less expensive, faster, more connected, and *smarter* than the last. On the one hand, the use of telemedicine among health professionals comes with the promise of revolutionizing the organization of health care, which can now be accessed increasingly at a distance. On the other hand, data-driven applications and new quantification practices seem to foster a belief in greater autonomy for patients. Increasingly equipped, connected, and measured patients are the active figures of an empowered generation that is becoming digitally engaged in their health by collecting, sorting, interpreting, and deleting many types of health and environment–related data [1]. Although these two examples seem to refer to very different realities, they illustrate a common fact that goes beyond their provisional and circumstantial distinctions: the supply of new digital technologies is only growing in the field of health, whereas it is challenging health care practices. For the sake of this argument, we will use the term *digital health innovations* to refer to the adoption of both professional-centered telemedicine or telehealth technologies (eg, teleconsultation, tele-expertise, telemonitoring) and patient-centered telecare technologies (eg, eHealth, mobile health, u-health, self-monitoring, self-help).

However, providing interesting technologies does not necessarily lead to their adoption or normalization. Some are successfully integrated into everyday practices, while others are used only by the circle of the first experimenters. What makes some of these innovations work? Which factors explain their success or failure? These questions are at the core of a very large body of multidisciplinary literature dealing with the issues of integrating new technologies into existing organizations and care practices [2]. This literature provides a whole set of criteria for assessing the chances of a successful integration [3]. Although it promotes macroscale analysis, it can sometimes overlook the actual practices and their concrete realities as presented to users. The purpose of this paper is to formulate a theoretical framework to better account for practice-related criteria in explaining the success of new health technologies. First, we should present and contextualize the work that we are mobilizing within the existing literature. Earlier studies conducted on telemedicine in the 1990s point mainly to the technical difficulties in explaining its slow diffusion. This focus suggests that it is sufficient to resolve these difficulties for telemedicine to be widely accepted [4]. Numerous studies conducted from the point of view of their acceptability or usefulness develop the idea that practices could be transposed as they are within the new framework. In this regard, May et al [5] state that “the existing literature on telemedicine [in 2001] has for the most part taken as its primary focus the utility and efficacy of the technology itself, as it is applied to particular clinical settings and problems. This is primarily clinical literature that is about establishing the safe practice of medicine using a diverse set of communications technologies.” This clinical approach, which seeks to evaluate telemedicine devices according to the principles of *evidence-based medicine* [6,7], is further coupled by numerous economic analysis models built around their cost-efficiency. However, it has since become clear that the development of innovations depends on much more varied and, more importantly, socially defined factors than merely the technical, clinical, or economic efficiencies. As such, these one-dimensional approaches tend to underestimate the importance of the institutional, organizational, and professional contexts in which these technologies are concretely integrated.

In this regard, many recent studies have focused on socio-organizational factors to assess the likelihood that a new technology will be integrated successfully into existing health care organizations and practices. Some works propose combining different socio-organizational aspects within a multidimensional assessment model [8]. For example, this is the case of the *Model for Assessment of Telemedicine*, which has been established in this field [9]. Although such models do indeed consider multiple aspects in the evaluation of digital health innovations, they are still far from capturing the plurality and complexity of the activities that need to be performed to make these technologies work concretely, on a daily basis, for very different actors who pursue highly varied objectives, in widely differing sociotechnical configurations. In fact, as shown by Pols [10] and, more broadly, by a large corpus of work in *Science and Technology Studies*, it is difficult to accurately predict the success or failure of innovations in telemedicine, nor the concrete integration modalities of digital technologies in existing health care practices. Users do not necessarily turn to these, out of a taste for technology (ie, technophilia). Moreover, a majority integrate them in a context full of uncertainty, where it is unclear how technologies will transform their practice. The only thing that can be predicted in this regard is the unpredictability of the adoption modes of these technologies. This unpredictability is based on the diversity of user profiles (eg, medical doctors, nurses, patients, helpers) and the types of technologies (eg, professional tools, connected objects, web platforms, and mobile apps). It is also based on various places (eg, hospitals or private practices, in cities or in small towns), sociotechnical environments (eg, resources, tools, and information systems), medical situations (eg, routine care, chronic disease monitoring, or emergency), and broader health care contexts (ie, during a health crisis) in which they are integrated, accessed, and used [11].

To understand the success of digital health innovations, it is necessary to examine the efforts or, more precisely, to quote Nicolini [12], “the work to make it work” provided by different stakeholders, not only by innovators and promoters but also by users, both health care professionals and patients. We suggest that all this *user work*, most of which remains invisible, is an important factor in explaining not only the phenomena of adoption of innovations but also the way in which they transform existing organizations and care practices. Here, we understand the notion of innovation as a continuous and evolutionary process in which users (and their practices) play a key role in the successful integration of new technologies.

### Scope and Purpose

Sociological studies have shown that the adoption of new health technologies by users is neither straightforward nor given [13]. As soon as a new technology is integrated into a care pathway, it inevitably faces resistance and creates obstacles due to the wide range of changes introduced in the existing context. These changes can concern the social and spatial organization of health care, the division of medical and paramedical work, the interactions between their various actors, the work activities themselves, as well as the knowledge levels and professional identities. To understand the success or failure of digital health innovations, it is necessary to pay attention to this prior context, which is bound to evolve with the integration of new technologies. More precisely, it is necessary to evaluate the adjustments that users must do to make it work by adapting the pre-existing context and sometimes by altering or even inventing new ways of doing things [14]. These adjustments represent a relatively constrained form of activity. However, without these, new technologies are difficult to normalize or can be abandoned.

A large body of work conducted in *Science and Technology Studies* provides valuable insights on the importance of these activities. This is done specifically by the Actor-Network Theory, which brings to light the unsuspected work of *translation* that must indeed be performed by innovators, both industrials who design and distribute new technologies and coordinators who seek to integrate them into their organizations [15-17]. Beyond traditional project management work, promoters must be able to *align* heterogeneous elements into a coherent whole. They must synchronize not only sociotechnical environments [18] but also practices and interactions that new technologies may suggest organizing in different ways. This alignment refers to work that is often invisible [19]. It requires building meaning (ie, *sensemaking*) and trust around these devices [20] and negotiating collective understanding among various categories of actors, including physicians, nurses, care assistants, patients, and family caregivers. These actors have *a priori* different interests that must be considered and converge to a minimum degree in order to support the normalization of new technologies. This is an eminently delicate task, not only because it requires articulating a variety of interpretations and modalities of action but also because new technologies affect autonomy, which is traditionally very important both for health professionals [21] and for their patients [22]; thus, it is difficult to negotiate.

These studies draw attention to the work that innovators and coordinators need to do to address the organizational complexity of integrating telemedicine devices in existing health care organizations. They also point out the *real work* needed to be done for these technologies to work on a daily basis, not only by promoters but also by users [19,23]. To make digital health technologies work, they must perform a series of additional activities that can be studied as a specific form of work [23]. Thus, to integrate new technologies into existing health care organizations, it is necessary to be able to measure *a priori* what is being asked of users [24]. In this regard, many approaches attempt to assess the acceptance of new technologies through functional, cognitive, or ergonomic analyses of their uses. However, on their own, these approaches are also struggling to produce a conceptual model whose ability to reflect the reality of practices depends on its interdisciplinary nature. A model that allows to do just that is a specific branch of sociology known as *practice studies* (or *practice-based studies*), which has resulted from the dialogue between multiple approaches, including not only *Science and Technology Studies* and innovation studies but also ethnomethodology and theories of distributed cognition that include more contributions of cognitive sciences and ergonomics [25]. Despite their differences, these disciplinary approaches all call for examination of how practices are engaged in and considered by the users themselves in their localized contexts. Inspired by these practice-based studies, this paper explains the success of digital health innovations through an analysis of the concrete activities that users, both health professionals and patients, must perform to make them work in a daily routine in their various socio-organizational contexts. Its purpose is to introduce the concept of *users’ invisible work* and to develop a sociological framework for studying it in the case of very different technologies and across several dimensions.

## Methods

This paper uses a three-part method to develop a sociological framework for studying *users’ invisible work*. First, it includes a thematic literature review that was previously performed and published by one of the coauthors. In this review, Alexandre Mathieu-Fritz organized and discussed major themes emerging from the existing literature conducted by both French and Anglo-Saxon social scientists on digital health innovations integration into existing care organizations and practices [2].

The second part of the method is to extend this review to introduce definitions and applications of *users’ invisible work* concept, which is coined at the crossroads of three conceptual models, each of which offers one keyword and one original work that serves as a starting point for building a framework. The first one is the *invisible work* model [26,27] used in the sociology of innovation, *Science and Technology Studies*, and sociology of work, to shed light on all efforts that players must deploy for an innovation to be successfully integrated in the existing organizations. The second model is that of *patient work* [23] coined in the sociology of professions and medical work, to recognize the key role of patients in their own health care. The third model is developed in a French corpus of sociology of work and economic sociology [28] and discussed in internet studies [29]. It refers to the unrecognized activities of consumers and web users that create value for private actors, both manufacturers and digital platforms. The purpose of this extension is not so much to present all these existing works as to identify the original contributions of these respective conceptual models in the study of different kind of work activities that users have to do to successfully integrate new technologies into their routines.

The third and last part of the method consists of producing a theoretical framework to study variations in the users’ work according to different types of digital health technologies and users. This framework is built using empirical work performed by the co-authors themselves in three different fields, each of which refers to a particular configuration of digital health practices. It includes teleconsultations between health care professionals and patients (in dermatology, geriatrics, and mental health), tele-expertise between health care professionals (in dermatology), and self-monitoring by patients themselves (monitoring of diabetes and cystic fibrosis). The framework was thus constructed by putting different practice-based criteria identified in the first part of the method, in the thematic literature review, through the filter of these three very different cases. This paper does not present the results of these studies, which will be published separately. However, it relies on this empirical work to generate an analytical grid that can be applied to study work activities in very different technological configurations.

## Results

### Overview

*Users’ invisible work* concept emanates directly from the perspective of the interactionist sociologist Anselm Strauss who coined the term *patient work* in the early 1980s. We first present the *patient work* model and its different applications in recent works on the use of digital health technologies by patients. We then extend this original definition not only to all technology users, both patients and professionals, but also to all kinds of work activities that remain invisible, including those related to more recent data-driven tracking apps. On the basis of this extended framework, we present an analytical grid that can be used both as an analytical tool in a research protocol and as a research agenda to assess the successful integration of digital health innovations into the existing health care organizations. The research protocol provides a tool to analyze this work through four dimensions related to the integration of new technologies (ie, organization of care, interaction between professionals as well as professionals and patients, clinical practice, and subjective experience of these users). As an agenda, it proposes to study the *users’ invisible work* in very different technical configurations and socio-organizational contexts in which it is performed. This qualitative research package is expected to reveal different types of work activities as more subtle criteria to explain variations in uses according to users' specific contexts and to produce a comparative analysis between different type of work that various digital health technologies need them to do.

### The Patient Work Model: Old Concepts, New Realities

In his work on medical worlds, Strauss drew attention to the fact that health care activities cannot be performed without the active participation of patients who have to perform a series of practical and cooperative tasks on a daily basis outside of health care facilities [23,30,31]. At first glance, it may seem peculiar to describe these activities as work. Generally, they are not considered as such, either by patients or health professionals [32]. However, this is justified in the case of chronic illnesses, which introduce profound changes in patients’ daily practices and experiences [33]. Furthermore, these activities can be expected from chronic patients, and their key roles can be even recognized by professionals. According to Strauss, patients are central actors in the division of medical work [34]. They have to develop certain corporal attention and organize the daily management of their care (ie, *illness work*). Beyond concrete activities, they also have to develop different forms of reflexivity to reconfigure their inner experience of the illness [35], meaning the way they view and build their future lives (ie, *biographical work*).

In traditional care, *technical* tasks, which cover the care practices undertaken for the direct purpose of altering the course of the pathology, are separated from *other forms of work.* Among these, Strauss identifies *clinical safety work* (ie, anticipation, verification, evaluation, and, where necessary, correction), *machine work* (ie, maintenance, monitoring, use and, where necessary, repair), *comfort work* (ie, aiming to reduce pain or discomfort), *information work* (ie, requests, reports, and reassurances), and *sentimental work* (ie, improving emotions of patients and coping with the psychological effects) [31]. These different forms of work are profoundly affected by the integration of new technologies that displace the concrete efforts as well as bodily sensations and thoughts that underlie the management of the illness [36,37]. This implies new forms of task delegation where patients take charge of their own medical surveillance and become true *diagnostic agents* [38]. In the case of telemedicine, Oudshoorn observes, for example, a certain disciplinarization of patients who often end up establishing *self-management techniques* [19]. Hence, they must perform a certain number of diagnostic procedures, acquire new skills related to the use of devices and the interpretation of symptoms, and, *in conclusion*, translate this learning into practical knowledge that they can use in the daily care of their disease [39].

These observations are supported by recent research on data-driven self-tracking apps. For example, the work of Mathieu-Fritz and Guillot conducted on the case of diabetes and self-monitoring devices [40]. The authors illustrated how the use of this device introduces new forms of work, reflexivity, and self-knowledge associated with the illness experience. Through a comparative analysis of old and new glucose meters, the authors showed that the permanent sensor placed on the arm alleviated some of the usual constraints posed by the fingerstick capillary testing methods [40]. Each of these devices refers to a set of constraints that organize the illness experience and its daily management differently. These constraints are not only physical (ie, pain) and material (ie, maintenance and transport of equipment) but also organizational (ie, anticipation and planning), spatial (ie, conditions for use of the devices), symbolic (ie, disclosure of the illness), and social (ie, strategies of discretion and breaking of interactions). This comparison of different types of patient work reveals more subtle criteria for understanding variations in the use of continuous glucose readers: more discreet and rapid, they can be used in social spaces where glycaemia measurement was not previously practiced, allows for more frequent measurements to be taken, and develop a different approach to anticipating treatment. The different forms of activities that the devices allow to perform or avoid transform the reflexive work of patients and, thus, the way in which they administer their own care. These observations are supported by several studies conducted more broadly in the field of prevention and well-being, where new self-quantification practices are being developed [41]. The confrontation of individuals with a *quantified self* not only provides new information to guide individual choices in the management of care but also produces new representations that profoundly affect the subjective experience of the body, the illness, and its daily management [42].

The application of this old concept of patient work to new self-monitoring and self-care practices points to the reflexive nature of new connected devices that are used more frequently in the medical field. The very nature of this work is changing in accordance with the new principles of visibility and recognition of these activities [43]. That said, one must wonder to what extent the action of scanning a sensor, or, even more unconsciously, the action of leaving mobile apps programmed by default, such as Apple's health watch, to collect and record data, can be studied as new forms of work. This debate, which is at the center of current concerns on the development of digital platforms, renews the interest and heuristic value of this concept to understanding how new *reflexive technologies* [44] affect the organization, practice, and experience of care [45,46]. From a critical perspective, some denounce the tendency of *techno-utopic* discourses to obscure the social, cultural, political, and economic dimensions of self-care technologies. Far from being neutral, these are, in fact, caught up in power relations [1,47]. This idea has been extensively developed in research inspired by *Science and Technology Studies*, which reports on the use of self-tracking devices as a reconfiguration of the physician-patient relationship through a new set of activities to generate and interpret data [48]. Further studies on daily data transmission devices to health care professionals show, for example, that patients can negotiate to redefine the objectives associated with the device by developing unexpected uses to prevent data transmission [49]. The merit of practice-based studies reveals itself through these works that study self-quantification as a particular form of work that must be performed *on* and *with* data [50], not only to contextualize them but also to articulate them with other forms of knowledge that supports the daily care practices [51]. This may represent one of the reasons why it is necessary to extend the analytical framework not only to various users (including health professionals) but also to different dimensions of their work, to include their reflexivity.

### An Extended Framework for Studying Users’ Invisible Work

Patient work is a powerful concept for highlighting and recognizing patients’ role and engagement in their own care. However, an extension is necessary to use it in the assessment of digital health innovations that requires considering all users, not only patients but also health care professionals who must make these devices work during their daily practice. For instance, Oudshoorn shows through the case of telemonitoring that physician not only interpret electrocardiograms but also take on a series of tasks unforeseen by the designers. They assist and reassure patients in the use of medical technology, ease concerns, and contribute to building trust in medical technology [19,52,53]. For the author, this is akin to *affective work*, which is equivalent to the *sentimental work* in Strauss’ classification [54]. Here, the notion of user work is clearly different from that of usability. The study of this work is not so much about the capacity or efficiency of uses as about the set of adjustments that users must make to resolve different types of conflicts that these technologies can introduce in the existing context. In that sense, studying users’ work is first, to understand how the integration of new technology transforms existing work activities.

In that regard, research have shown that new digital health technologies do not simply equip practices from *outside* but contribute to transform them in a profound way, from the *inside*. Nicolini’s work is particularly instructive in this regard. By *zooming in* on the practices and *zooming out* on the broader organizational context [55], the author highlights how work is redistributed among practitioners, as well as with artifacts (ie, *nonhumans*). In this context, the adoption of these devices depends on a series of learnings, settings, and adjustments that must be made in users’ working practices [13]. A typical case is that of teleconsultations during which the roles played by each participant will be different from those usually played in a face-to-face setting [56]. Similar to other scholars, Nicolini observed that professional practices were transformed in situations where new forms of task distribution and delegation occur not only between physicians but also between physicians and paramedical professions (ie, nurses and caregivers) [57]. In some cases, important tasks that are considered to be central in the professional practice (ie, *true work* [58], as opposed to *dirty work* [59], which is considered to be peripheral) can be delegated to patients themselves.

These new forms of cooperation can also be observed among actors from different specialties who are placed at different hierarchical levels. In these configurations, they can contribute to the sharing of medical and clinical knowledge and enable certain delegates to *increase in expertise.* Moreover, it can also redefine the boundaries of their professional territory and identity [60]. Research on telemedicine devices show that “the question is not so much what the new activities allow, but rather to what extent they allow existing and appropriate forms of professional knowledge and practice to be put in place” [5]. However, this observation must be nuanced. Professionals may also get round the specific constraints of telemedicine (eg, physical distance and deprivation of *sensory inputs* [19,61]), for example, to develop new therapeutic techniques in the context of teleconsultations in mental health, by testing sooner than usual their *clinical intimacy* or by asking questions more frequently [62]. Thus, the integration of new technologies may end up producing new practices. Here, the technology itself becomes a full actor with whom one, not only redistributes existing work but also jointly produces new information that needs to be interpreted. These new forms of work become just as apparent to patients, for example, in the case of quantification devices that introduce new indicators in self-monitoring practices.

The patient work model must be extended to include not only existing work activities that have to evolve with the integration of new technologies but also new activities that users must perform and skills they need to acquire to use these technologies during their daily activities. Thus, the study of users’ work invites a more systematic look at the articulation and coordination of different types of tasks within the socio-organizational context in which technologies are being integrated and used. The work of Mathieu-Fritz et al sheds light in this respect. First, the authors show that coordinating physicians play a key role in solving difficulties and problems that the designers did not foresee [52]. The authors also highlight different types of *framing* work with the purpose of establishing rules and guidelines and ensuring that users become autonomous [63-65]. Technical framing aims to teach how to use devices effectively (eg, synchronized use of mobile camera, dermatoscope, and spirometer), whereas social framing is about establishing ways to interact efficiently during teleconsultations (eg, presenting oneself precisely and systematically, speaking louder, and articulating better). Clinical framing defines the protocols to follow (eg, patients’ clinical history, auscultation, diagnosis, prescriptions, or indications), whereas organizational framing involves cultivating appropriate attitudes and behaviors (eg, being punctual, completing forms and, reminding appointments), to ensure that all actors are present at a given time on both sides of the camera.

The authors are particularly attentive to the additional coordination activities that medical and paramedical actors must perform for the day-to-day operation of these devices [27]. Referring to the work of Strauss, they emphasized the importance of *articulation work* [66,67]. In their research on the medical world, Strauss et al [68] define the social organization of care as a *negotiated order* that results from the constant efforts of the actors to produce, often informally, an agreement on the best ways to organize tasks daily. As Star [26] reminds us, this articulation work is beyond the scope of rational work organizations. It consists of organizing, coordinating, and combining different types of activities conducted by various actors (eg, a clinical examination, an x-ray, a blood or blood pressure test, prescription, and administration of medication) to ensure that “the staff’s collective efforts add up to more than discrete and conflicting bits of accomplished work” [31]. The integration of new technologies has transformed coordination work. New coordination tasks are also required to ensure day-to-day operation. These activities are important, even sometimes decisive, so much so that certain works identify their intensity as a rejection factor of new devices [69].

Studying all this background work, most of which remains invisible, is crucial to account for the efforts required to make technologies work. However, most of these work forms are difficult to account for in the design and integration process of new technologies [70,71]. On the basis of the lessons learned from this literature, we propose to define the scope of the *users’ invisible work* concept as all the concrete and reflexive activities that both health care professionals and patients perform to make digital health technologies work within their daily routines around health care. One of the original contributions of this framework is to emphasize the importance of reflexivity and experience forms that develop through local and subjective confrontations with the technologies in the trajectory of these innovations. The notion of reflexivity is used here in its two registers. Retrospective reflexivity refers to the ability to return from experiences and past events. Similar to immediate reflexivity, lessons learned from previous experiences serve to restructure action as they unfold [72]. The reflexive dimension is becoming increasingly important with data-driven apps, either because this work is increasingly automated by connected and intelligent objects, to the point that this is done sometimes entirely out of awareness or, on the contrary, because the data are increasingly visible to the patients who produce, interpret, and communicate it. In any case, one of the main contributions of this extended framework concerns precisely the way it suggests analyzing the work activities as they are performed by users, but also, and more importantly, their reflexivity (ie, what they think and feel as they perform them). It is only then that some nuances can be explored to understand resistance to technology. This is the case for some health professionals who reject new technologies not because they resist change but because they believe that these do not allow them to do what they consider to be the core of their work, their *real work* [73]. In other words, rejection can express a transposability issue where users consider that they cannot work or care with the same quality they would usually expect to have. This is clearly a separate issue from a social resistance to change.

### A Sociological Research Package to Assess Digital Health Innovations

This theoretical framework can be used both as an analytical tool in a research protocol and as a qualitative research agenda to assess the chances that digital health technologies have to be successfully integrated into existing health care organizations. In a research protocol, for any given technology, it proposes to produce an overview of different actors who constitute the sociotechnical network around their use. All user types (eg, physicians, patients, and family caregivers) and material supports (ie, technical objects, instructions, and protocols) must be fully included as human and nonhuman actors, which must be aligned to some extent for innovations to be successfully integrated into daily routines [17]. This ecological approach must also be applied to cover a variety of tasks and activities without which innovation is difficult to develop or abandoned. In this regard, all forms of work (eg*,* articulation work, patient work, and information work) and their various definitions (eg, well-done or acceptable work, desired or satisfactory work and prevented work [74]) must be considered. Overall, the goal is to capture the diversity of the actors performing a range of different tasks and activities, both practical and reflexive in nature, to integrate new technologies into existing health care organizations (Textbox 1).

**An ecological approach to the diversity of digital technologies, users, and work.**

**Types of technology**

- Equipment sets (telepresence station, teleconsultation booth, telemedicine trolley, etc)
- Connected medical tools (spirometer, stethoscope, otoscope, ultrasound scanner, electrocardiogram, dermatoscope, etc)
- Mobile apps (crowdsourcing, quantification, gaming, self-help, etc)
- Web-based platforms and software services (networking, forums, videoconference, etc)
- Connected objects (sensors, readers, automatons, robotics, AI, vocal assistants, chatbots, carebots, wearables, etc)

**Types of users involved**

- Health professionals (specialized or general practitioners, nurses, etc)
- Allied health professionals (paramedics, therapists, assistants, auxiliary personnel, social workers, etc)
- Patients (chronic, nonchronic or acute, emergency, etc)
- Healthy individuals (eg, sportsmen and women)
- Family caregivers (parents, partners, friends, etc)

**Types of tasks and work**

- Translation work (eg, alignment and sensemaking)
- Coordination work (eg, articulation, framing)
- Patient work (eg, illness work, sentimental work, and information work)
- Users work (concrete efforts such as taking measurements, ticking boxes, or sharing data, but also different reflexivity forms)

To do so, this framework investigates the circumstances under which this work is performed through four dimensions related to the integration of new technologies (ie, organizational, interactional, practical, and experiential). The first dimension, organizational, concerns coordination modalities of health care. The term organization refers to the physical and material environment in which digital health technologies are integrated and whose level of saturation can be an important factor in users’ rejection. It also refers to all the additional coordination activities that users must perform, such as articulation work that realigns affected *lines of work*, and framing work that defines rules to normalize uses. This organizational dimension relates to broader changes in the care pathway due to new technologies that define the scope and composition of work groups differently. This is, for example, the case of teleconsultation that allows health care professionals to be brought together with different specialties and sometimes actors who were not participating in traditional consultations, thus contributing to the formation of new micro–work collectives and to the evolution of patient care pathways [60,65].

The second dimension is that of interactions between various actors within health care organizations and with patients. It draws attention to the place of these actors in the whole system of interactions that is mobilized around patients’ care [75]. It first includes new modalities in which these actors interact with each other through new technologies that tend to open up the singular colloquium between the physician and patient and change the relationship between the two by adding new actors, both from the medical side and the patients’ side. In this regard, Oudshoorn refers to a major transformation in the geography of responsibilities [76] based on a new spatial and temporal distribution of activities related to care delivery. In this *techno-geography*, new proximities may develop between patients and health care professionals [77]. Oudshoorn insists on the way in which the *digital proximity* established through new telemedicine devices highlights “protocol-driven communication, daily surveillance, and self-care” [52] as full-fledged dimensions of the interactions that forge remote care relationships. As it is, this new type of proximity sets aside the psychosocial dimensions of traditional face-to-face care, which requires considering extramedical aspects. These transformations also occur at a more symbolic level. For example, some studies show that hierarchical representations or expert legitimacy can be at stake between professionals who use digital health technologies. Some can associate the use of certain technologies with a lack of professionalism. For example, some mental health professionals tend to hide their teleconsulting practices, which are not well regarded in their professional environment [62]. These symbolic effects also concern patients whose use of these technologies can vary according to the social environment in which they have to make them work. This is the case for diabetic patients who have to use a glucometer that not only discloses their illness but also disrupts social interactions in which they can be engaged [40].

The third dimension of user work is clinical practice, that is, activities directly associated with the diagnosis and medical treatment of patients. It includes new forms of distribution and delegation of tasks between physicians and allied professions [57] and with patients [19] that may enable sharing medical and technical knowledge and increasing expertise. Some users may also end up working twice as much to integrate these devices (ie, patient’s double work) [40]. Furthermore, this redistribution of tasks transforms the broader topography and temporality of care activities, both for the professionals and patients. In this regard, Nicolini showed that the daily exercise of telemedicine is characterized by a *stretching out* of sociomaterial arrangements in space-time and consequently an *expansion* of health care activities [57]. This means that these reconfigurations go far beyond the simple spatiotemporal redistribution of existing activities. They contribute to transforming the social spaces in which these activities are performed. Oudshoorn showed how the homes of older adults who are hospitalized at home with the help of telemonitoring devices becomes a hybrid place in which conflictual logics of care and aesthetics can coexist [76]. This new geography profoundly affects the object and content of these activities. It transforms the relationships between health care professionals by redistributing their work differently, sometimes with artifacts.

The fourth dimension is the subjective experience of these users, which refers to their feelings and representations that they develop within and through their practice. In fact, the integration of new technologies alters not only concrete work activities during the clinical examination but also professional judgments involved in the formulation of a diagnosis. For instance, building trust seems to be a core issue for health professionals who develop an *experimental attitude* [40] toward these devices. As mentioned above in the case of teleconsultations, professionals who engage in such practices with an unclear status question the extent to which previous practices can be transposed into the new framework. For example, they consider the problem of accountability for encountered difficulties. They also consider the costs of this transposition in economic (related to the equipment that needs to be acquired and their compatibility or interoperability with the existing ones), organizational (related to the time and resources needed for preparation), cognitive (related to learning and training needs), and social and symbolic (related to the image conveyed by the device) terms. This is not so different for patients whose subjective experience also plays a key role in how they manage their own health care. Indeed, the experience of illness encompasses the patient's work, but goes far beyond it. It refers to the whole inner experience of the disease, to all that is felt (eg, bodily sensations) and thought (eg, what one would like to do, or, on the contrary, to avoid) subjectively. Overall, the experience of the disease refers to the social definition of oneself (eg, when one tries to hide one's illness and when one talks about it or shows it in some way). New technologies also challenge these reflexive activities. An example is given by Van Hout in his work on a telemonitoring device that has been used with homecare patients who were minimizing their symptoms or even omitting to tell palliative care nurses [37]. However, the device that allowed patients to assess the severity of their various symptoms on a scale of 0 to 5 has not always worked, as patients have criticized the device for reminding them of symptoms they did not yet have or were at risk of having (ie, display effect). Therefore, *forgetting* the device, and thus the disease, can become an important factor in their adoption by patients [78].

This paper proposes to study *users’ invisible work* through these four dimensions. Table 1 presents these practice-based criteria in a chart that can be applied as a tool in research protocols. However, there is no single recipe or exclusive way of applying this grid, which is rather a collection of criteria that can be combined as needed, depending on the technologies, contexts of use, and users being studied.

**Table 1:** An analytical grid to study users works through its four dimensions.

Users’ work and criteria		Application examples
**Organizational configurations**		
	Ecology of artifacts	Interoperability between information systems; unforeseen problem-solving; degree of saturation as a factor of rejection
	Additional coordination work	Framing work (social, technical, clinical and organizational) to establish the rules of use; intensity of the articulation work as a rejection factor
	Recomposition of the care pathway	Scope and composition of new work collectives; forms and conditions of preventative actions
**Interactional settings**		
	System and modalities of interaction	Opening of the singular colloquium of patient-physician to new actors; digital proximity with the patients
	Forms of cooperation	Delegation of tasks and sometimes even “real work” to other professionals and artifacts; patients as diagnostic agents
	Symbolic effects	Professionals' legitimacy among colleagues; patients' illness in their social environment
**Clinical practices**		
	Topography and temporality	Duplication of the therapeutic space; “expansion” of health care activities; new constraints that organize patients’ reflexive work
	Learning or cognitive aspects	Knowledge barriers; “increase in expertise”; double work of patients; disciplinarization of patients
	Consideration of the psychosocial aspects	Reduced in digital proximity
**Subjective experiences**		
	Commitment and trust in fuzzy practices	Experimental attitude; Transposability issues; economic, organizational cognitive, social and symbolic costs of transposition; Accountability problems
	“Forgetting” about the disease or device	New information produced by the device (display effect); representations that affect the experience and the daily management of illnesses

Furthermore, this framework can also be applied as an agenda that calls for further research on the integration phase of various digital health technologies into work systems (eg, connected devices, data collecting and displaying apps, and more infrastructural telemedicine projects, such as teleconsultation cabins). The purpose of this agenda is to redefine some of the *a priori* distinctions (ie, telehealth vs digital health) through variations that can be observed at the level of the different work forms that require users to accomplish in very different configurations. This agenda should include studies of technologies that are used in different spaces, both very localized and spontaneous ones, such as teleconsultations, and mobile and ubiquitous ones, such as self-tracking devices. This agenda should also contain studies on apps in which data can be more or less important to explore the various effects of reflexive technologies on the self-knowledge of patients associated with their illness experience. It must cover devices used in different medical conditions, for example, for rare and common pathologies, to identify how technology adoption modes vary with the rarity of a disease. A preliminary list of such variables is presented in Table 2, which aims to cover a very wide range of configurations, including spatiotemporal, technical, social, and medical variations, to develop this comparative approach between different work forms required in the context of their uses.

**Table 2:** Variables in the use of digital health technologies.

Variables			Description
**Spatial-temporal**			
	Topography	Single place (eg, fixed teleconsultation cabin), semi mobile (eg, telemedicine trolley), multiple place (eg, mobile apps)	
	Frequency and punctuality	Frequent (eg, multiple times a day) or rare (eg, once a month), precise (eg, on appointment) or approximate (eg, each morning)	
	Duration	Continuous (eg, wearable devices and sensors) or punctual (eg, teleconsultations)	
**Technical**			
	Autonomy	Automated (eg, parameterization) or triggered actions (eg, synchronization)	
	Visibility	Visible (eg, reader) or invisible (eg, implants)	
	Connectivity	Autonomous (eg, pedometer) or connected devices (eg, smart watches)	
	Artifactuality and agentivity	Cognitive (eg, stocking data) or interactive (eg, following feeds), passive (eg, visual representations) or active (eg, alerts and notifications)	
**Social**			
	Context of use	Routine check-up (eg, blood work), emergency (eg, allergy crises) or well-being (eg, sport diet)	
	Modality of use	Individual (eg, self-help, measurements, etc.) or collective (data sharing applications, dyadic or triadic teleconsultations, etc)	
	Type of actors	Professional caregivers (interaction with physicians, nurses, etc) or family members (assistance by parents, friends, etc)	
**Medical**			
	Pathology	Chronic (eg, diabetes) or acute (eg, heart spasm), rare (eg, cystic fibrosis) or common (eg, kidney disease)	
	Specialty	Primary care (eg, general medicine), secondary care (eg, specialists), tertiary care and hospitalization (eg, surgery)	
	Type of treatment	Preventive (eg, dietary programs), curative (eg, physical therapy), palliative or hospice (eg, cancer treatment)	

With this agenda, it becomes possible to produce a comparative study of users’ work variations in the case of different digital health practices and explain the success or failure of new technologies, according to more or less data-driven, real-time, frequent, or visible nature of activities they require users to do.

## Discussion

### Principal Findings

This paper draws attention to the plurality of most invisible and unrecognized tasks and activities that need to be performed by professionals and patients to make these devices work in various contexts. As a result, it proposes a theoretical framework that allows the assessment of digital health innovations by studying these work activities under different settings and the transformations they introduce through four main dimensions: organizations, practices, interactions, and experiences. For any given technology, it can be used as a tool in a research protocol to study concrete work activities performed by users to integrate them into their daily lives. It can also be applied as a research agenda that covers a wide range of technological configurations to develop a comparative approach through practice-based criteria.

### Main Contributions

This theoretical framework makes three main contributions to the literature. First, it reports on professional practices and patient experiences jointly and in an articulated way. Second, it seeks to guide analytical practices by further operationalizing the theoretical approaches of practice-based studies in a methodological framework to help better understand the successful integration of new technologies into existing health organizations. Third, it redefines traditional categories such as *technology acceptance* or *resistance to change*, through an analysis that remains more faithful to the activity and the concrete reality of the users themselves.
